# Navigating open-source waters: the pharmaceutical industry’s role in bioontology development

**DOI:** 10.1093/database/baaf066

**Published:** 2025-09-30

**Authors:** Shawn Zheng Kai Tan, Joshua Daniel Valdez, Saritha Vettikunnel Kuriakose

**Affiliations:** Novo Nordisk A/S, Novo Nordisk Park 1, 2760 Måløv, Denmark; Novo Nordisk A/S, Novo Nordisk Park 1, 2760 Måløv, Denmark; Novo Nordisk A/S, Novo Nordisk Park 1, 2760 Måløv, Denmark

## Abstract

Bioontologies are core to many data management strategies, artificial intelligence and machine learning initiatives, and search functionality within many pharmaceutical companies. Despite their integral role, many bioontologies, along with their associated tools, are maintained predominantly by academia and their partners, government supported initiatives, and the general community. In this comment, we will dive into some of the reasons behind this trend and argue that there exists a mutual advantage for the life science industry, and pharmaceutical companies in particular, to actively contribute to the advancement of public ontologies and open-source tools. This benefit extends beyond ethical and moral considerations and aligns with strategic interests. Additionally, we will explore practical approaches for contributing, sharing our (Novo Nordisk’s research and early development) experience in doing so.

## Introduction

Ontologies are core to many data management strategies. They enable, at least in part, every aspect of Findable, Accessible, Interoperable, and Reusable (FAIR) data principles [1]. They enhance findability by offering a semantic structure that facilitates complex querying. Moreover, they can provide controlled vocabulary to promote data interoperability, and facilitate a clear understanding of the data, fostering reusability. Bioontologies are complex artefacts that also serve as a knowledge base to leverage the collective wisdom of the community. For example, the Gene Ontology [2] is commonly used as a method to analyse gene set enrichment [3]. Due to the inherent complexity of biology and ontologies, maintaining such artefacts requires substantial curation efforts involving parties well-versed in both subjects. Despite their prolific use in industry, the development, curation, and maintenance of open-source bioontologies have predominantly been managed by academia and their partners, government supported initiatives, and the general community. Most tasks are often undertaken within projects that share an interest in their development, exemplified by initiatives such as the Monarch Initiative [4] and the BRAIN Initiative Cell Atlas Network (BICAN) [5]. These research initiatives typically focus on specific objectives unrelated to the development of ontologies and are often time-limited, and ontology development is done as a means to an end rather than the focus. This often leads to curators having to prioritize project-oriented tasks over the ongoing maintenance or improvement of public bioontologies. This in turn leads to situations, where curatorial efforts are predominantly done only to address immediate project needs rather than proactively managing the ontologies to fulfil user requests. As examples, Uberon [6] and Cell Ontology [7][8] have anywhere between 200 and 300 open issues. These numbers are despite the automated processes that close issues if they remain inactive for 18 months. Overall, this scenario depicts a community operating on scant resources, burdened by a substantial backlog, and lacking financial incentives. In this comment, we propose that the pharmaceutical industry (pharma) in particular, and life science industry in general, can assume a more substantial role in the development and maintenance of open-source ontologies, which would be a strategic investment in FAIR data management and AI powered drug discovery.

In the next sections, we discuss three ways in which pharma can practically engage and discuss some strategic advantages of doing so. Acknowledging the difficulty in instantiating an abstraction, we also share our [Novo Nordisk Research and Early Development (R&ED)] experience. Our aim is to encourage ontology teams within industry to consider this comment as a call to action, inviting them to contribute to the ongoing efforts in developing open-source bioontologies, where possible.

## Contributing to ontology content

Bioontologies function as knowledge bases for continuously evolving fields. As such, effective bioontologies are not fixed artefacts, instead, they dynamically evolve in tandem with the advancement of knowledge. This implies that continuous development and maintenance are necessary. Furthermore, given the complex nature of bioontologies, issues in content (e.g. duplicated terms that need to be merged, over restrictive logical axioms, missing information, and so on) are almost inevitable and require correction. While many ontologies have automated QC (quality checks) and a review system for changes, there will inevitably be things that slip through the cracks.

As avid users of these ontologies, pharma are well-positioned to unveil potential issues. Moreover, the practical applications within pharma provide a compelling impetus for ontologies to house the most up-to-date information and corresponding models. For example, as public data sources tend to utilize up-to-date ontologies, having the most up-to-date ontologies in-house means better interoperability with external data sources. Additionally, given bioontologies also function as knowledge bases, having the most updated ontologies would also provide us the ability to keep current with newly incorporated knowledge. When it comes to addressing issues or making additions to ontologies, pharma has two avenues—rectifying at the source or addressing in-house. There are instances where in-house remedies are essential, especially when dealing with proprietary information, such as drugs in development. However, for issues that do not involve proprietary information, like identifying duplicate terms, refining hierarchies, or incorporating nonproprietary terms such as anatomical parts, we contend that investing the effort to rectify these at source is worthwhile. A practical first option of rectifying at source would be that instead of addressing issues in house, fixes can be reported to the developers/maintainers of the ontology. Many ontologies have ticketing systems that allow users to request new terms or changes, and report errors. Ideally curators will then pick these up and address them. However, as mentioned above, most public ontologies are resource strapped, and tickets may take a long time to fix (or in cases become abandoned). Therefore, an alternate and perhaps more sustainable option is to own the change by making the fixes, making requests into the repository, and seeing the ticket all the way through until it is merged and published. This, however, requires that the in-house team has both the bandwidth and capability to do so. A second way to contribute to the public efforts would be by participating in community efforts in mapping between ontologies, and other resources (like databases, thesauri, and so on). Mapping in this context refers to harmonizing identifiers for equivalent concepts between resources. While good mappings are not essential in ontology development *per se*, they are highly important in data integration, which is one of the key uses of ontologies in the biomedical space. Contributing to mappings therefore greatly increases the useability of the ontologies. Projects like biomappings [9] provide ways of doing so, and there are initiatives to develop frameworks for the same like the one spearheaded by the European Open Science Cloud [10].

Fixing at source has a few strategic benefits compared to fixing in-house. First, and probably the most important and compelling reason would be the reduced burden of maintenance. Ontologies are dynamic artefacts and are constantly updated as knowledge evolves. In-house fixes therefore must be applied each time ontologies are updated. Furthermore, it will be necessary to maintain a set of coherency checks to ensure any changes made in-house do not clash with changes made in the source public ontologies. Second, making changes at source ensures better interoperability with external data. This is not without its challenges. As each ontology has its own quirks, significant technical and domain knowledge will probably be required to make pull requests to the source ontology. For example, each ontology has specific procedures on how obsoletion of terms are done, from annotations or tags used to mark obsolete terms and their replacements, to notice periods and procedures. These quirks can also extend to more technical quirks, like how ontologies import terms from other ontologies (e.g. through importing modules of subgraphs or importing a merged product). If these pose a large challenge to a team, opting for opening tickets and leaning on ontology developers could be a way to navigate these complexities. However, this approach is likely to have slow turnarounds that might not be practical for the fast pace needed. Fortunately, this challenge is reduced by the fact that a significant number of the ontologies used in the biomedical sphere utilize the Ontology Development Kit (ODK) [11], which provides standardized structure, workflow, and tooling. By learning a single workflow, users can initiate pull requests themselves, thereby expediting the process. Another potential challenge is that competitors may be able to derive research directions from the changes made in public ontologies. While we believe this is highly unlikely, steps can be taken to be conservative in how you approach this (keeping certain concepts as in-house enrichments for example), which is especially easy to manage in a precompetitive environment. At Novo Nordisk R&ED, we have in-house specialists trained in the ODK and ontology editing to aid in pushing our needs to public ontologies. In our case, we have recruited professionals already trained in ODK, who have subsequently provided training to our other ontologist who already possess foundation skills and therefore only need to learn the new technical framework. While this process requires a dedicated time commitment for training, publicly available resources have proven valuable for developing our internal training materials, allowing us to customize content to suit our requirements, such as deploying the ODK on AWS EC2 instances. Importantly, we believe that even without hiring specialists in ODK, other teams can effectively adopt these workflows by leveraging available resources. As a starting point, the obook has lessons plans known as pathways that structures tutorials, how-to guides, explainers, and references structured in a way that can systematically train someone on how to edit many Open Biological and Biomedical Ontology (OBO) ontologies (https://oboacademy.github.io/obook/pathways/ontology-curator-go-style/). The obook also contains a full lesson on contributing to OBO ontologies for users who prefer a workshop style lesson (https://oboacademy.github.io/obook/lesson/contributing-to-obo-ontologies/). The Monarch Initiatives further supplements this with ongoing training sessions on different topics in ontology development (https://oboacademy.github.io/obook/courses/monarch-obo-training/). There are also slack communities (e.g. obo-communitygroup.slack.com) that teams can join to get more support and keep in close contact with developers and maintainers of ontologies.

Our proactive approach to enhancing the quality of these ontologies is evident in our commitment to addressing errors we identify. We prioritize fixing these errors in the source ontologies, and at the time of writing, we have reported 20+ issues to various ontologies and related resources, 11 of which we have fixed by making pull requests. These issues include fixing textual definitions, hierarchies and logical axioms, and merging duplicated terms. This has resulted not only resulted in a more robust ontology product (e.g. from merging duplicated terms or adding logical axioms), but also a more user-friendly ontology product (e.g. in splitting parentage in logical axioms to make hierarchies more explicit), something that we believe that we as a pharma end-user are well suited to address.

## Contributing to open-source tooling

Ontologies can be challenging to develop, maintain, and utilize. Managing ontologies often require tooling at every stage. A few approaches to address this are building in-house, utilizing open-source tooling, or buying off-the-shelf products. These approaches are not mutually exclusive, and often, organizations use a combination of all three. While open-source tooling has limited utility in many companies due to the lack of professional support and lack of access controls, they are still useful in intermediary steps like processing and manipulating ontologies, utilizing and creating mappings, and parsing standardized shorthand like curies. In this section, we will advocate that contributing to the development of open-source tooling, can be worth the effort and time required, not just for the community, but also for the strategy of the company.

There are a few ways in which pharma can contribute to this endeavour. The first of which is to make a conscious decision to deploy said open-source tooling wherever possible in the ontology development/management workflow in conjunction with in-house developed solutions and/or vendor solutions. The considerations for use of open-source tooling differ between organizations and often include the security risks (e.g. standard IT risk assessment and AI risk assessment following the EU AI act) from deploying open-source code, scalability of solutions and support in case of critical failure. In addition, having inclusion metrics like active development and stable releases, developer access, and longevity of solution will ensure sustainability of solutions. For example, some open-source software might connect to public services that can track data (e.g. connecting to—OpenAI API), in which case, the organization needs to find an acceptable solution. Once a tool has been derisked and deployed internally, there needs to be a commitment to using the same despite the challenges. This translates to working with the developers of the open-source software to fix and improve it acknowledging the benefit of high technical expertise despite the time delays that could be involved. Another approach could be to fund open-source tooling to capitalize on collective knowledge from the community while contributing back to the same.

The organization, by contributing to the open-source tooling efforts, benefits from having access to the community to onboard new members into the niche teams (semantic teams are often isolated teams in pharma). For example, the aforementioned Monarch Initiative holds regular training that focuses on developing ontologies using open-source tooling like the ODK [11], ROBOT [12], OAK (https://github.com/INCATools/ontology-access-kit), and so on. Similarly, the aforementioned obook contains documentation in the form of tutorials, how-tos, and so on that can be used to onboard users.

The added benefit of engaging with the open-source community is that tooling is not only created by a small group of developers, but instead, a community is available for bouncing ideas, honing code, and developing robust solutions. When tools are developed by a single individual, there can be significant inefficiencies, such as outdated coding practices that make onboarding difficult for new contributors, particularly when documentation is sparse. Additionally, relying on a single developer can create a bottleneck, especially if that individual leaves the organization, jeopardizing the continuity of development. One of the biggest advantages in using open-source tools are that they are not at the mercy of single developers and their preferences, hence developing more resilience. Moreover, if the organization aims to ensure that workflows are interoperable with external data, using open-source tooling is advantageous. For example using SSSOM tooling enforces SSSOM standards [13] for mapping that are widely used in the bioontology sphere. This ensures that pipeline inputs or outputs adhere to these standards, enabling interoperability with other SSSOM mappings. Although the use of open-source SSSOM packages is not required to achieve this, the benefit of already having existing tooling provides strong impetus to utilize it rather than developing it in-house.

At Novo Nordisk R&ED, we have utilized open-source tooling in our pipelines wherever derisking was possible [14]. For example, we utilize ROBOT [12] to develop application ontologies from public ontologies, and we use SSSOM tools and standards in our pipelines to map ontology concepts. Where we lack the expertise or resources, we have opened tickets. For instance, as users of curies package (https://github.com/biopragmatics/curies), we have shared a challenge with functionality which others have agreed with, and has eventually been fixed by the maintainer (https://github.com/biopragmatics/curies/issues/63).

## Connecting with the community

Ontologies are by nature collaborative, representing the collective knowledge and influence of the community. This is especially so for public reference ontologies, where the scope is wide, and resource limited. As users of public bioontologies, pharma has a direct interest in the development and evolution of these frameworks. Effective engagement in the ontology community requires dedicated resources and commitment based on the needs of the organization. From our experience, the type of engagement could range from providing strategic inputs and thought leadership to sponsoring resources. The channels for engagement also differ between different communities and often require an understanding of the landscape. For instance, public ontologies often host their discussions on git repository platforms like GitHub, where active issue tracking provides an immediate forum for ontology-related discourse. While these repositories are a logical starting point for engagement, broader conversations often unfold in more fluid environments that may be less visible. Accessing these wider discussions usually involves platforms such as Slack channels, which either requires an invitation, or knowledge on where to find the link. One practical approach to gaining entry is to initiate contact with the ontology developers. An alternate approach would be to join the OBO community slack channel, which is available at https://obofoundry.org/docs/participate.html. For a more structured entry point into the community, organizations such as the OBO Foundry or the Pistoia Alliance offer opportunities to foster meaningful engagement.

Return on investment in such engagements is often neither obvious nor directly relatable to drug discovery in pharma. Articulating the value of this investment to leadership can be daunting and the challenge is amplified by the typically slow pace of community-driven initiatives.

We contend that, beyond the intrinsic value of contributing to the community whose resources are leveraged (quid pro quo), active participation yields strategic advantages. While the popularity of genAI has resulted in increased attention to ontologies in pharma, this development is still nascent. Often semantics and ontology teams are a very small part of the pharma workforce, and such teams benefit greatly from community engagement not only to keep abreast of latest technology but also to spar on organization specific challenges. Such platforms also allow to advocate for specific interests which may in turn be shared by other organizations in the same sphere enabling crowd sourcing of innovative solutions. For example, we contribute to the thought leadership shaping the Pharma General Ontology (PGO), an initiative led by the Pistoia Alliance to unify key concepts across the pharmaceutical landscape. This collaboration between the participating pharmaceutical companies and academic institutions has enabled the effective use of frameworks, such as LinkML (https://linkml.io/). Engaging with experts from various teams has also facilitated streamlined workflows that benefit not only our organization but also the broader community. For example, utilizing existing frameworks like LinkML allowed us to utilize existing community developed tooling around it (e.g. converting the semantics part of the LinkML contract into an owl application ontology), hence accelerating our development process. Similarly, the codevelopment of a data contract that utilizes LinkML to unify key concepts and allow easier transmit of data operationalizes LinkML into a new framework.

This creates a symbiotic dynamic where the community benefits from user-driven feedback, while the team can influence the trajectory in ways that are advantageous for themselves. Furthermore, immersing teams in their respective communities exposes them to new perspectives from peers across different teams and disciplines, fostering an environment where challenging the status quo is the norm. For example, our work with the PGO mentioned above in developing ways to transmit data between pharmas has led to us utilizing LinkML as a framework for embedding semantics into data contracts. This is now in the process of being adopted and operationalized in our organization.

At Novo Nordisk R&ED, we also actively participate in the OBO Foundry, where we have representation in the operations committee. Additionally, as highlighted in previous sections, we maintain a dynamic presence in the repositories for the ontologies we utilize, acting not just as consumers, but also as contributors to the evolution of these critical resources.

## Conclusion

Pharma often shies away from community engagement due to challenges related to confidentiality of content and pace of community-based development. While these challenges are real and acknowledged, we also believe that thoughtful engagement in community driven bioontology development provides a strategic advantage as highlighted in the earlier sections. There is a rift between the bioontology community and pharma, one that needs to be healed by pharma going beyond being consumers. We suggest some practical ways for pharma to get involved in the community, based on our experience. Beyond our suggestions, there are other practical ways that pharma can support the development of bioontologies and the communities around it. First, simply citing relevant ontologies, projects, and databases in publications, presentations, and conferences can greatly help these resource show their value and secure resources. Acting as industry partners and providing letters of support for grant applications similarly can strengthen funding opportunities for ontology projects. Alternatively, pharmas can directly fund precompetitive ontology initiatives either directly by contracting academic workers through joint projects, or through organizations like the Pistoia Alliance. For example, development of the bioassay ontology was supported by the Pistoia Alliance SEED project that was funded by pharmas. Lastly, selecting suppliers and vendors that promote the adoption of open ontologies and standards where sensible will greatly help in their adoption and create a more unified and interoperable framework across the industry and academia. Overall, we consider this a call to action, a springboard for wider discussions and catalyst for other pharma to join us in this journey. Our hope is to nurture a cohesive bioontology community, represented by all parties with an interest in its development, in a symbiotic relationship.
